# A disorder in executive functions crosses traditional diagnostic borders of the schizophrenia-bipolar spectrum

**DOI:** 10.1192/j.eurpsy.2022.412

**Published:** 2022-09-01

**Authors:** I. Szendi Md Habil, P. Pajkossy, A. Bagi, M. Marián, Á. Szőllősi, M. Racsmány

**Affiliations:** 1 Kiskunhalas Semmelweis Hospital, University Teaching Hospital, Psychiatry Unit, Kiskunhalas, Hungary; 2 Research Centre for Natural Sciences, Institute Of Cognitive Neuroscience And Psychology, Budapest, Hungary; 3 University of Szeged, Department Of Psychiatry, Szeged, Hungary; 4 Budapest University of Technology and Economics, Department Of Cognitive Science, Budapest, Hungary

**Keywords:** Transdiagnostic, RDoC, Executive functions, WCST

## Abstract

**Introduction:**

Our series of studies in the spectrum of psychosis (schizophrenia, bipolar affective disorder, schizoaffective disorder) is based on the concept of the RDoC system.

**Objectives:**

In this study, we were interested in knowing whether cross-diagnostic disturbances in cognitive functions can be found in the spectrum and whether they predict clinical symptoms.

**Methods:**

In the study, N = 66 schizophrenic (M = 38.2 ± 9.37 years, 26 women), N = 30 bipolar (M = 47.4 ± 9.35 years, 19 women), N = 33 schizoaffective (M = 39.8 years± 11.3 years, 21 women) and N = 28 healthy subjects (M = 36.5 ± 9.9 years, 14 women) participated. All subjects underwent the Wisconsin Card Sorting Test (WCST), Raven Test, Digit Span Test, Visual Patterns Test, Letter and Semantic Fluency tests, Metaphor and Irony Comprehension, Directed Forgetting, Stop Signal Test, and Lexical Decision Task. In addition, symptom rating scales were administered (PANSS, SANS, YMRS, MADRS).

**Results:**

Based on our results, the performance of the WCST-deficient group lagged behind the WCST-non-deficient group and the healthy control group in most executive control tests. Importantly, this effect was independent of diagnosis, so it appeared in all three patient groups. Members of the deficit group had a higher rate of negative symptoms.

**Conclusions:**

Disruption of executive functions is a transdiagnostic feature of the schizophrenia-bipolar spectrum, which could be associated with any diagnosis.

**Disclosure:**

No significant relationships.

